# Data description of “City boundary and urban district boundaries, Vienna, 1920”

**DOI:** 10.1016/j.dib.2021.107382

**Published:** 2021-09-20

**Authors:** Ulrich Kral, Emre Can Sönmez, Ferdinand Reimer

**Affiliations:** a Technische Universität Wien, Institute for Water Quality and Resource Management, Karlsplatz 13/226, Vienna 1040, Austria; b Unaffiliated, Vienna, Austria

**Keywords:** Historic GIS, City boundary, Urban district boundaries, Digitalization

## Abstract

This article presents geospatial datasets for administrative boundaries of the city of Vienna in 1920. One dataset covers the city area and another the urban districts. The boundaries were retrieved from historic analog maps that show the course of the borders at this time. GIS software was used to geocode the analog maps and construct the polygon-features for the city and 21 district areas. These datasets are useful for mapping the spatial coverage of administrative units in the 1920s and to group and analyse further historic GIS data.

## Graphical abstract

**Figure:**
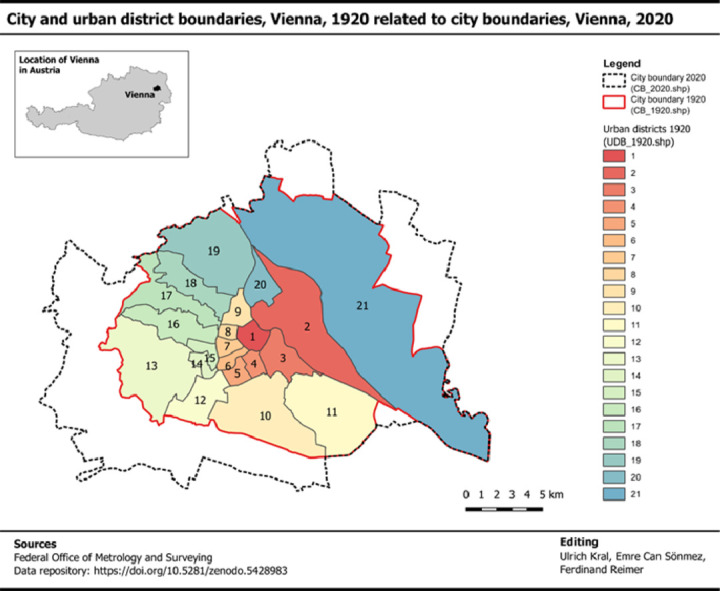

## Specifications Table

**Table:** 

Subject	Geographical Information System
Specific subject area	Administrative boundaries
Type of data	Geospatial datasets
How data were acquired	The creation of the geospatial dataset is based on desktop work. The data were acquired by vectorizing boundaries from analog maps.
Data format	Secondary data
Parameters for data collection	The key parameter is the geographic coverage of (i) the city area and (ii) the urban district areas of Vienna in the year 1920. Vienna is the capital of Austria (Latitude: 48° 12′ 30.56″ N, 16° 22′ 19.49″ E) [1]. The conditions for data collection consider the timestamp and the spatial accuracy of the administrative boundaries in historic analog maps. We used analog maps that (i) represent the situation in 1920 and (ii) that allow to reconstruction the course of the borders.
Description of data collection	The data inputs were collected from data repositories and archives as detailed in the next section.
Data source location	Data inputs: • Analog map: Vienna and surroundings 1938-46 [2] • Analog map: Urban district maps 1925-30 [3], [4], [5], [6], [7], [8], [9], [10], [11], [12], [13], [14], [15] • Analog map: Building age map 1920 [16] • Dataset: Administrative borders 2020 [17] • Dataset: Street graph 2020 [18] • Dataset: City map 2018 [19] • Dataset: Data records from Statistical Yearbook 1929 [20] Data input descriptions can be found in Table 3.
Data accessibility	Repository name: Zenodo Data identification number: 5428983 Direct URL to data: https://doi.org/10.5281/zenodo.5428983 Directories: • City boundary 1920 • Urban district boundaries 1920 • Background data

## Value of the Data

• The geospatial datasets include administrative city boundaries of Vienna in the year 1920. These datasets are important, because it's the first time that the boundaries are available in a machine-readable format. They can be processed with geographical information systems.
• Urban city planners, environmental historians and geographers, who need historical data on the administrative boundaries, profit from the dataset.
• The dataset can be used to (i) group and analyse historical data by the urban districts of 1920, (ii) to georeference scans of analog maps based on the presented boundaries, and (iii) the data set can also be used, together with other datasets on administrative boundaries, to analyse the spatial changes of administrative boundaries over time.

## 1 Data Description

**Fig. 1:**
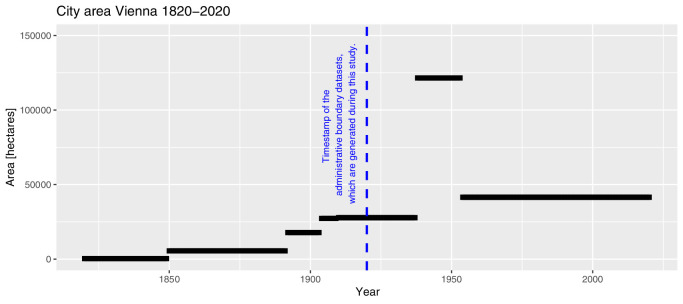
City area of Vienna from 1820-2020. Data source: City of Vienna [21].

The administrative boundaries of Vienna changed multiple times in history. On their website, the city administration presents a timeseries with the number of districts, the city area and the length of the city boundary as well as the reasons for changes between 1849 and 2021 [21]. According to the website, the city covered about 360 hectare (ha) until 1849. The city incorporated suburbs and surrounding areas in 1850, 1892, 1904, 1910 and 1938. After World War 2 (1938-1945), in 1954, Vienna was limited to its's current size of 41,487 ha. The timeseries of the city area from 1820-2020 is presented in Fig. 1.

**Fig. 2:**
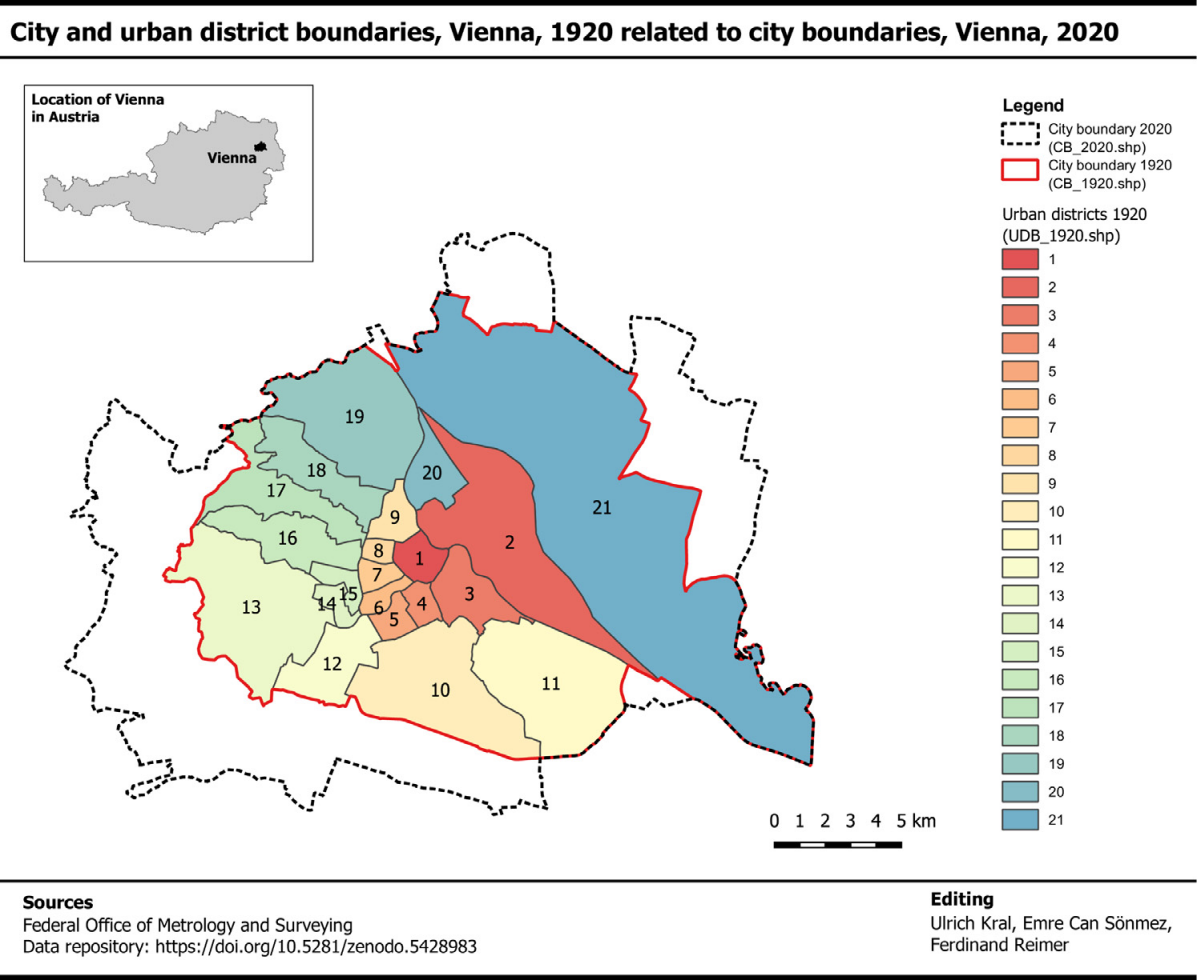
Administrative boundaries of the city of Vienna including the city boundary in 1920 and 2020 as well as the geographical coverage of urban districts in 1920. It is noted that the boundaries of 1920 were created during the study and the boundary of 2020 was retrieved from Federal Office of Metrology and Surveying [17].

From a mapping point of view, the cities’ administrative boundaries became machine-readable with the introduction of computers from about 1980 onwards. Before this time, administrative boundaries were mapped on paper. The spatial information is not available in a machine-readable format and cannot be processed with geographic information systems (GIS). This is a barrier for processing historical data. Against this background, this article presents geospatial datasets with administrative boundaries, namely the city and the urban district boundaries, for the city of Vienna (Fig. 2).

We have selected the timestamp 1920, which represents the situation immediately after the end of the Austro-Hungarian Empire (1867-1918) [cf. 22]. During this period, the administration collected and published a wide range of statistical data on urban district level, which can be spatially visualized with help of the urban district boundary dataset from this article (see section “value of the data”).

With respect to the temporal scope of the dataset, it is noted that the city area was constant from 1904 to 1937 [21]. So, we assume that the course of the city boundary was valid in this period. With respect to the urban district boundaries, we found indications of boundary changes between 1904 and 1937. For instance, parts of the suburb “Strebersdorf” were incorporated into the 21st district Florisdorf, which changed the area from 9,496 to 9,914 ha [23]. The areal statics also shows differenes of more than 10 ha for the districts 2, 13, 19, 20 and 21 between the periods 1907-1910 and 1910-1923 as well as for the districts 2, 20 and 21 between 1910-1923 and 1923-1927. The differences might be caused by revised computation methods or by changes in the course of urban district boundaries within the city boundary. Within the scope of this study, we didn't investigate any spatial changes in the urban district boundaries between 1904 and 1937. Consequently, the urban district boundaries, which are presented here, are valid at least for 1920.

This article documents the generation two geospatial datasets, namely the city and urban district boundaries of 1920, which are specified as followed:

• *City boundary 1920.* The CB_1920.shp includes 1 polygon-feature with a total area of 27,697 ha. This polygon covers the area of the city in 1920. The dataset includes 2 data fields (Table 1) and 1 data record.

**Table 1:** Attribute table structure of CB_1920.shp file.

Field	Name	Comment
FID	Identifier of polygon-feature	This field includes a unique identifier for the polygon-feature.
Shape_Area	Area of polygon-feature	This field specifies the city area in hectares.
• *Urban district boundaries 1920.* The UDB_1920.shp file includes 21 polygon-features, each standing for an urban district in 1920. The total area of the polygon-features is 27,697 ha and therefore identical with the area of the polygon-feature in the CB_1920.shp file. The dataset includes 3 data fields (Table 2) and 21 data records.

**Table 2:** Attribute table structure of UDB_1920.shp file.

Field	Name	Comment
FID	Identifier of polygon-feature	This field includes a unique identifier for the polygon-feature.
UDN	Urban district number	This field specifies the urban district number with reference to the urban district distribution.
Area	Area of polygon-feature	This field specifies the city area in hectares.

It is noted that the corresponding data repository at Zenodo [24] also includes background data to reproduce the geospatial datasets CD_1920 and UDB_1920 as well as the figures in the article independently from the provision of the input data by the data providers. The background data cover an geospatial dataset with the city boundary of 2020 [17], line-features that were extracted from geospatial datasets with cadastral community borders and street courses in 2020 [17,18], as well as georeferenced scans of historical maps [2,16].

It is also noted that a preliminary draft version of the city boundary 1920 was used in Fig. 2 of the article “Buildings schematic of Vienna in the late 1920s” [25]. In this article, we used the layer to communicate the geographical coverage of another dataset with 42,861 data entries on buildings [26].

## 2 Materials and Methods

### 2.1 Data inputs

Six data inputs were used to create the CB_1920.shp and UDB_1920.shp file (Table 3). One the one hand, we used scans of analog maps that show city and/or urban district boundaries as present in 1920. On the other hand, we used current geospatial datasets as a reference for georeferencing the scans of the analog maps and for re-using the line-features to reconstruct the administrative boundaries of 1920. One addition data input, namely the Statistical Yearbook 1929, was used to validate the data outputs (CB_1920, UDB_1920). All input data are public available and can be downloaded from the web.

**Table 3:** Data input description. Notes: “SHP” = Shapefile, “TIF” = Tagged Image File, “JPG” = Joint Photographic Experts Group file, “PDF” = Portable Document Format file.

Title	Description	Format	Reference including download link
Vienna and surroundings map (1938-46)	The analog map shows the city boundaries valid before 15. October 1938, from 1938 to 1945 and from 1954 onwards. The historical context and details on the map content are provided by Wien Geschichte Wiki-Contributers [27]. Acronym: VSM	TIF	[2]
Building age map (1920)	This analog map shows an (low-resolution) overview of the city and urban district boundaries in 1920. Acronym: BAM	JPG	[16]
Urban district maps (1925-30)	The analog map sheets show the city and urban district boundaries by urban district at high resolution (scale: 1:1,500) for the second half of the 1920ies. They are available for 14 of 21 districts (1-3, 7-13, 17-20). With respect to the timestamp, the Municipal and Provincial Archives of Vienna [28] dated one set of maps (signature 3.2.2.P10/2.119833) with “around 1920” and the second set of maps (signature 3.2.1.1.P4.317./2) with “1930”. It is noted that both sets include identical maps and the maps show social housing complexes, which indicates the same publication year of both set of maps and a timestamp between 1925 and 1930 [29]. Acronym: UDMs	JPG	[3], [4], [5], [6], [7], [8], [9], [10], [11], [12], [13], [14], [15]
Administrative borders (2020)	The geospatial dataset includes administrative boundaries, including city, urban districts and cadastral community boundaries of Vienna.	SHP	[17]
Street graph (2020)	The geospatial dataset includes the municipal and regional road network in Vienna. The network is represented by line-features that represent, in general, the center lines of the roads.	SHP	[18]
City map (2018)	The geospatial dataset, also called area multi-purpose map, includes polygon-features for building footprints in Vienna.	SHP	[19]
Statistical Yearbook (1929)	This yearbook shows the urban district areas and the city perimeter for the period 1910-1923 [30]. We converted the analog areal statistic into a machine-readable dataset and added the corresponding PDF page from the analog yearbook. Acronym: SY_1929	PDF, XLSX	[20]

### 2.2 Data processing

We used ArcGIS Desktop 10.8.1, created a new map and set the reference system / projection with MGI / Austria GK East (EPSG-Code: 31256, https://epsg.org). Next, we reconstructed the city boundary of 1920 and the urban district boundaries of 1920 (see section 2.2.1 and 2.2.2). The validation of the datasets is presented in Section 2.3.

#### 2.2.1 City boundary of 1920

The reconstruction of the city boundary of 1920 included three key steps.

First, we imported and georeferenced the scanned maps VSM and UDMs as followed.

• *Vienna and surroundings map (1938-1946)*: The VSM shows two boundaries of relevance for this study. On the one hand, the city limits before 15 October 1938, which in essence was also the course of the border in 1920. On the other hand, the border which was realized in 1954, which is still valid today. So, we georeferenced the VSM based on the analog city boundaries in 1954 [2] and the geospatial dataset of city limits in 2020 [17].
• *Urban district maps (1920ies)*: The UDMs show building footprints and streets by urban district as well as the urban district boundaries at high level of detail. We georeferenced the analog UDMs based on the analog street courses and the geospatial dataset with the street graph in 2020 [15].

Second, we used the ArcGIS function “Polygon to Lines” and converted the cadastral community polygon-features, which are part of the Administrative borders (2020), into line-features.

**Fig. 3:**
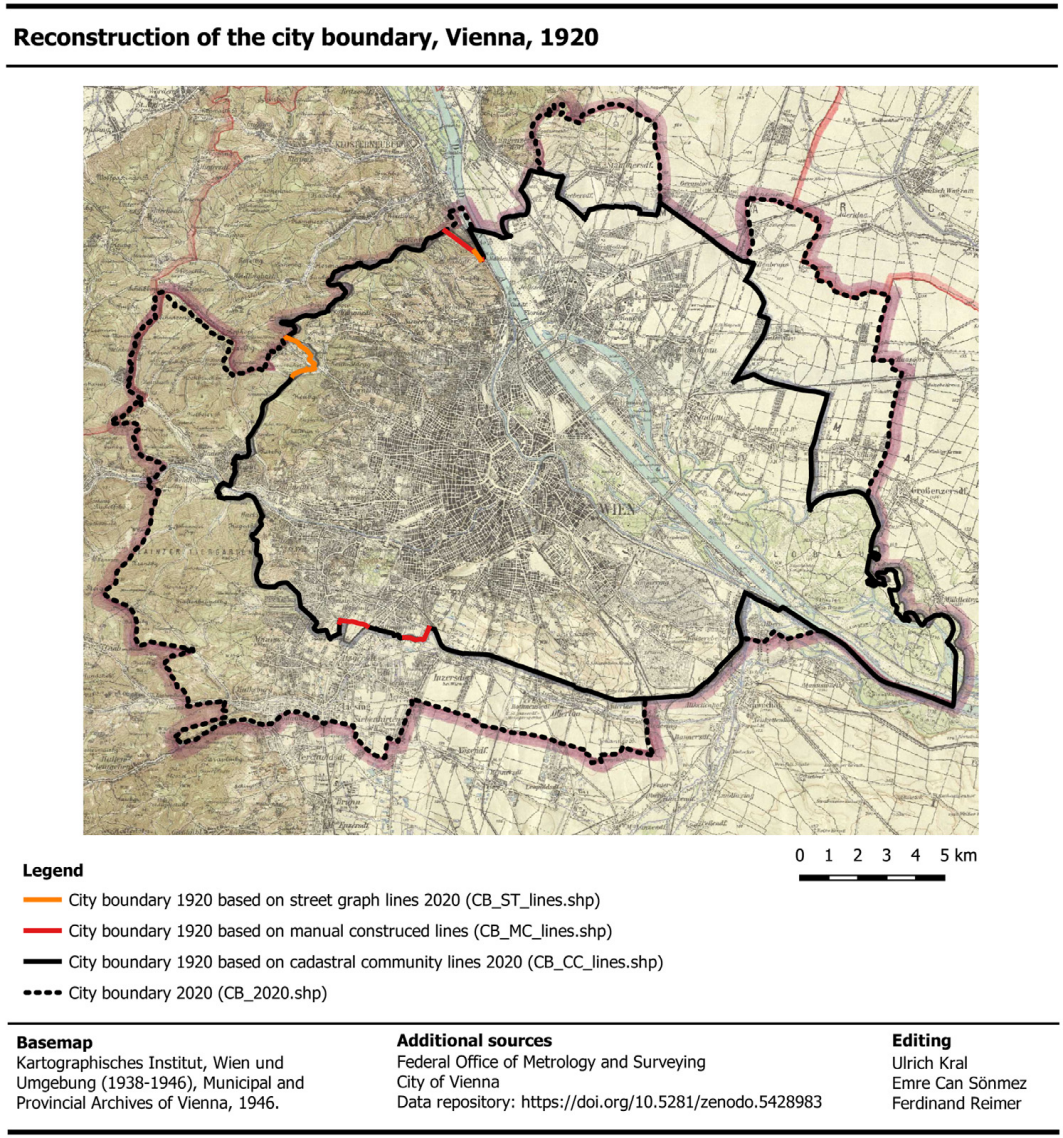
Reconstruction of the city boundary before 1938 based on the Vienna and surroundings map 1938-46 [2] and the geospatial datasets with cadastral community lines (black) taken from Federal Office of Metrology and Surveying [17], street graph lines (orange) taken from City of Vienna [18] and manually constructed lines (red). The city boundary 2020 was taken from Federal office of metrology and surveying [17]. It is noted that the datasets of this figure are included in the data repository at Zenodo [24, ../03_Background data]. (For interpretation of the references to color in this figure legend, the reader is referred to the web version of this article).

Third, we reconstructed the city boundary 1920 by visually comparing the location and course of the analog city boundary in respect to the location and course of the cadastral community boundaries 2020. With respect to the total boundary length, we found that 94% corresponded with the cadastral community lines of 2020 [17]. So, we added these line-features to the city boundary layer 1920 (Fig. 3, black lines). About 3% of the total length corresponded with the course of the street segments [18] and we copied these line-features to the city boundary layer 1920 (Fig. 3, orange lines). The course of these lines was confirmed by a cross-check with the corresponding UDMs of district 17 and 19 [5,11]. The remaining 3% matched neither the cadastral community lines nor the street lines. So, we manually constructed the lines (Fig. 3, red lines) based on the city boundaries as given in the UDM for the 12th and 19th district [11,31].

It is noted that Fig. 3 shows a short, red line-feature in the south of Vienna, which clearly deviates from the analog city boundary. In this case, we selected the boundary from the UBM of the 12th district [31] and the Generalregulierungsplan 1912 [32], respectively. We didn't find any map that validates the section of the boundary as shown in the VSM.

#### 2.2.2 Urban district boundaries 1920

The reconstruction of the urban district boundaries of 1920 included three key steps.

First, in addition to VSM and UDMs, we used the Building age map 1920, which includes a city-wide overview of the city and urban district boundaries around 1920. We georeferenced the overview image, on the top left of the scanned map [16], based on the geospatial dataset with city boundaries 1920 (CB_1920.shp), as described in Section 2.2.1.

Second, we added the geospatial dataset with building footprints of 2018 [19]. This map helped us to locate the course of the urban district boundaries between buildings (see next step).

**Fig. 4:**
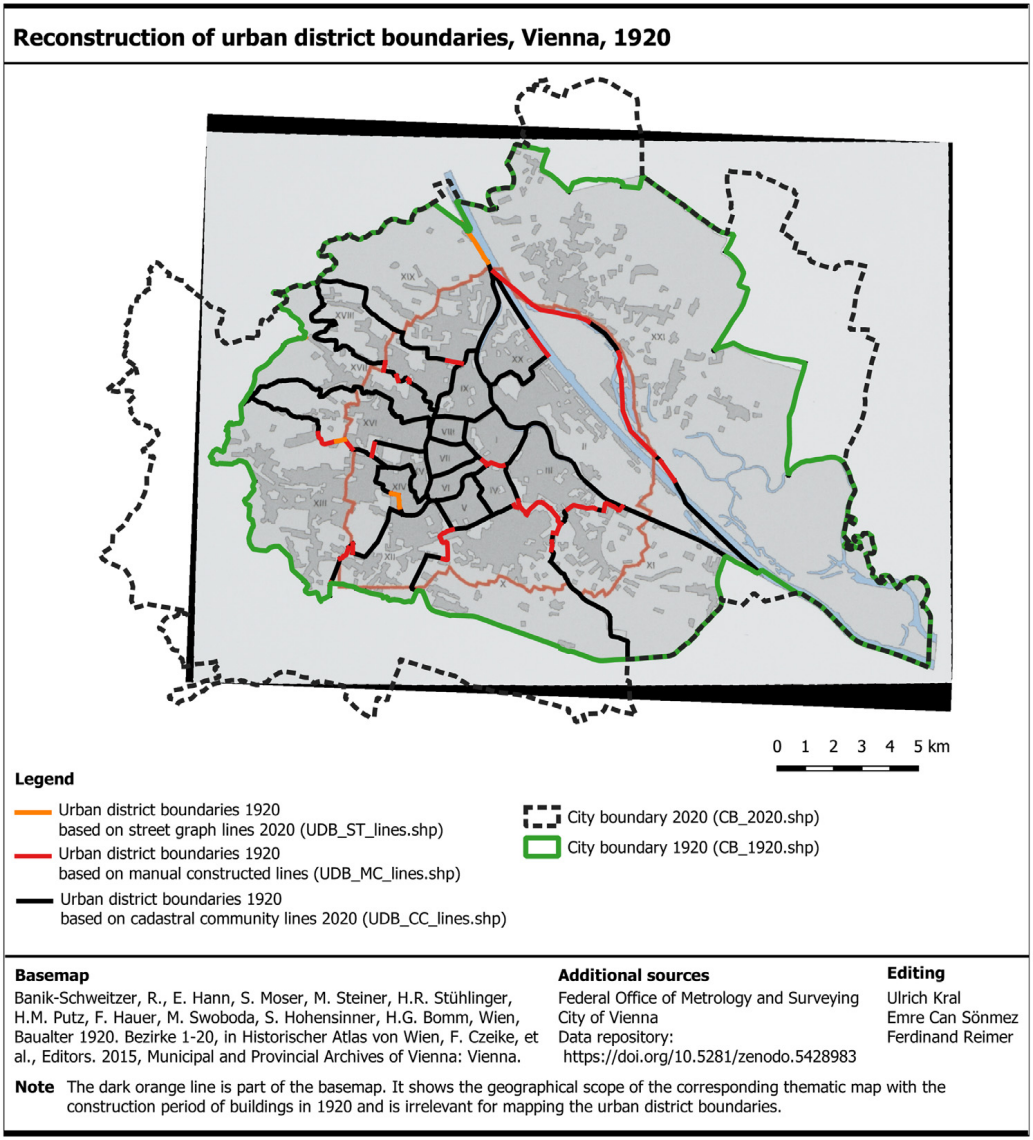
Reconstruction of the urban district boundary 1920 based on the building age map 1920 [16] and the geospatial datasets with cadastral community lines 2020 (black) taken from Federal Office of Metrology and Surveying [17], street graph lines 2020 (orange) taken from City of Vienna [18] and manually constructed lines (red). It is noted that the datasets of this figure are included in the data repository at Zenodo [24, ../03_Background data]. (For interpretation of the references to color in this figure legend, the reader is referred to the web version of this article).

Third, and in analogy to the reconstruction of the city boundary, we visually compared the location and geometry of the analog urban district boundaries from the BAM with the course of the cadastral community lines of 2020 [17]. We found that the urban district boundaries of 1920 follow the cadastral community lines 2020 to a wide extend. So, we copied these lines to the urban district boundary layer 1920 (Fig. 4, black lines). Only minor segments of the analog urban district boundary followed streets lines of 2020 (Fig. 4, orange lines). Segments that neither followed the cadastral community lines 2020 nor the street lines 2020, were manually constructed with line-features based on the analog boundaries in the scanned UDMs (Fig. 4, red lines). We also used the city map (2018) [19] as complementary visual information for mapping the course of the boundaries, because the UDMs also show the building footprints.

### 2.3 Validation of data outputs

This section use external data, from the statistical yearbook 1929 (SY_1929) [30], to validate the data outputs, namely the geospatial datasets with the “city boundary 1920” and the “urban district boundaries 1920”, based on city perimeter 1920, city area 1920 and the urban district areas of 1920, respectively.

#### 2.3.1 City perimeter 1920

The perimeter of the polygon-feature of CB_1920.shp is 102,10 km and the SY_1929 recorded a length of 101,10 km [20]. We didn't find any evidence for the difference, but it might result from imprecise mapping of the boundary in the analog maps, any deformation of the scanned maps during georeferentiation, or imprecise interpretation of the analog boundaries and their digital reconstruction. Having all the data uncertainties in mind that come along with the reconstruction of historic boundaries, we feel that difference of about 1% of the total length is acceptable and justifies the selection of the VSM as reference for digitalizing the boundary.

#### 2.3.2 City area 1920

We compared the city area between CB_1920.shp and SY_1929. The SY_1929 includes area records for urban districts in the reference period 1910-1923 [30] and is available in a machine-readable format at Zenodo [20]. The CB_1920 polygon-feature has 276.97 km^2^ and the SY_1929 recorded 278.08 km^2^, whereby the difference is 1.08 km^2^ (0.39%). This discrepancy might be due to an imprecise reconstruction of the boundary by us. Another reason could also be that the data records in SY_1929 are imprecise, because the areal estimation in the 1920ies might be less precise than those today. The course of the border could potentially be improved by using the geographic description of the course of the border as defined in state law on 19 December 1890 [33] and maps from the 21^st^ urban district that show the exact course of the city boundary. However, the data quality depends on the purpose of the data. The current data quality satisfies our needs to map historic data on urban district level in the future. With having this data in mind, we feel that the selection of the VSM as basemap is justified. Experts who need more precise data on the boundaries might use complementary maps and historic information.

#### 2.3.3 Urban district area 1920

**Fig. 5:**
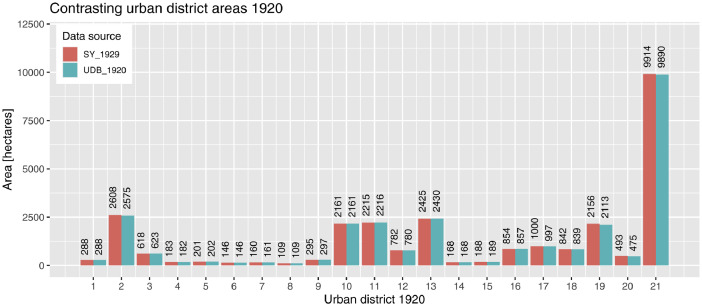
Contrasting the urban district areas 1920 between the geospatial dataset “UDB_1920” [24] and the Statistical Yearbook 1929 (SY_1929) [30].

The maps for the urban district distribution of 1920 and 2020 differ from each other due to expansion of the city and changes in boundary delineations. Whereas the boundaries of 2020 are geodesically surveyed and therefore precise, the boundaries of 1920 have been digitized from scans of analog maps and are therefore potentially imprecise. To make plausible the boundaries of 1920, we compared the urban district areas between UDB_1920 and SY_1929 [20,30]. With respect to UDB_1920, the area sum of all 21 polygon-features is 27,697 ha. On an urban district level, the polygon areas and the statistical records slightly differ from each other (Fig. 5). The largest absolute difference is 43 ha (−2.0%) in district 19, followed by 33 ha (−1.3%) in the district 2, 23 ha (0.2%) in district 21 and 18 ha (3.9%) in district 20. The difference in the other 17 districts is less than 5 ha per district. As stated in chapter 2.3.2, the data quality is adequate for the purpose of our research in the field. So, the scans of the UDMs are suitable, at least of our purposes, to generate the geospatial datasets. Experts who are interested in more precise boundaries, especially for the districts 2, 19-21 should look for complementary information sources to improve the data quality.

## 3 Usage Notes

The SHP and CSV files can be imported and processed with QGIS (https://www.qgis.org), ArcGIS (https://www.arcgis.com), PostGIS (https://postgis.net/), the R Project for Statistical Computing (https://www.r-project.org/) and any other tools that enable geospatial data processing.

## CRediT authorship contribution statement

**Ulrich Kral:** Conceptualization, Methodology, Investigation, Writing – original draft, Visualization, Funding acquisition. **Emre Can Sönmez:** Investigation. **Ferdinand Reimer:** Investigation.

## Declaration of Competing Interest

The authors declare that they have no known competing financial interests or personal relationships which have or could be perceived to have influenced the work reported in this article.
